# Categorization Activities in Norwegian Preschools: Digital Tools in Identifying, Articulating, and Assessing

**DOI:** 10.3389/fpsyg.2019.00973

**Published:** 2019-05-22

**Authors:** Pål Aarsand

**Affiliations:** Department of Education and Lifelong Learning, Norwegian University of Science and Technology, Trondheim, Norway

**Keywords:** digital literacy, children, categorization, ethnomethodology, guided participation, professional vision, digital competence, preschool

## Abstract

The article explores digital literacy practices in children’s everyday lives at Norwegian preschools and some of the ways in which young children appropriate basic digital literacy skills through guided participation in situated activities. Building on an ethnomethodological perspective, the analyses are based on 70 h of video recordings documenting the activities in which 45 children, aged 5 and 6, and 8 preschool teachers participated. Through the detailed analysis of two categorization activities – identifying geometrical shapes and identifying feelings/thoughts – the use of digital tools in the social organization of the activities is examined. The article finds that children’s digital literacy activities encompass visual, verbal, audio and embodied competencies that become relevant, and thus accessible for learning, in the interaction between the children and between the adults and children by serving as norms and guidelines for what constitutes correct categorizations (geometrical shapes and green and red feelings) in the situated activities, and that are appropriated and actualized by the children in interaction with their peers. The findings also show how the categorization practices in preschools deal with symbols and labels in ways that create and sustain socially organized ways of knowing, seeing, and acting upon the world. Digital media are embedded in routines, procedures, and socialites that are part of these categorization practices; they are part of how children are instructed to experience, interpret, understand, and act in the world. Moreover, the different technologies created different conditions for the children’s participation. It was found that peer interaction was part of the digital literacy activities that involved such mobile technologies as smartphones and tablets, while when using non-mobile technologies, e.g., smartboards, the activities were structured more as ‘classic’ classroom activities, primarily guided by the teacher and the didactic material presented through the smartboard.

## Introduction

Digital literacy practices have become an intrinsic part of Norwegian children’s life in preschool. Touchscreens and smart speakers are well-known examples (cf. McTear et al., 2016; The Norwegian Media Authority [Medietilsynet], 2018) of how the technological interfaces have changed and facilitated young children’s use of digital tools (Nacher et al., 2015; Price et al., 2015). When digital tools have become part of Norwegian preschools, they have generated digital literacy practices that include a range of activities such as reading, listening, touching, adjusting, curating and producing symbols and signs (cf. Lafton, 2012; Leu et al., 2017). The social and material environment is inherent in local definitions of what it means to know something, and what is considered relevant knowledge can be seen as dynamic and changing (Aarsand and Melander, 2016; Cannon et al., 2018). This points out that the meaning of action, symbols and signs is situated and thus closely related to the context in which they appear (e.g., Goodwin, 1994; Kress, 2000). This also tells us that knowing how to act can be seen as a *pragmatic resource*, where children and adults use it according to how appropriate, meaningful and useful it is in the particular activity (Gillen and Hall, 2012; Aarsand and Bowden, 2019). Thus, being a digitally literate person means being a member of a community where one is able to read and produce relevant action in line with what is expected from the position one occupies at the appropriate time and place.

In the present text I will examine how children participate in teacher-initiated activities where digital media are used as tools. Drawing on ethnomethodological and conversation analytic perspectives (e.g., Schegloff, 1996; Goodwin, 2000), I will ask the question: how are digital literacy practices constituted in interaction? The focus is on how children participate in various categorization activities and what resources and strategies they use to differentiate between geometrical shapes and different feelings.

## Digital Literacy in Early Childhood

Studies of digital literacy practices in preschools have shown how children and a preschool teacher use and interpret multimodal interactional resources in the production of shared understanding and meaning making of digital texts (Björk-Willén and Aronsson, 2014; Davidson et al., 2014, 2017; Bevemyr and Björk-Willén, 2016). In an Australian study of preschool children’s use of YouTube, Davidson et al. (2014, 2017) have pointed out that digital literacy practices and meaning-making processes involve embodied interactional resources as well as online texts, thereby transgressing the online/offline dichotomy (see also Marsh, 2014).

Digital literacy activities can be seen as situated in the sense that how participants understand and deal with signs, icons, symbols, gestures, pointing, colors, and images is closely related to what, where, when, and together with whom these occur. Aarsand and Melander (2016) found in a study of Swedish children’s digital literacy practices at home and at school that these activities encompass verbal, embodied, and social competencies. They also found that these competencies are accessible for learning in the interaction between adults and children by serving as norms and guidelines for what constitutes knowledgeable participation in media literacy activities. A study of Australian, Norwegian, and Swedish children’s digital game playing at home, preschool, and afterschool (Danby et al., 2018) found that children collaborated with their peers to advance the game by using multiple strategies such as instructing each other, monitoring each other’s action and problem solving (cf. Björk-Willén and Aronsson, 2014). Knowing how to participate in digital literacy activities at public venues, such as preschools, involves social competences where children learn from each other how to organize the activity, evaluate other participants’ way of acting, understand what is happening and know how to work with the tools. Bearing this in mind, it has been argued that participating in digital literacy activities involves understanding, using and acting according to social norms and expectations (Davidson et al., 2017). Moreover, it has been found that social norms for what counts as the ‘correct’ way to talk about digital texts and experiences, and what counts as competence, are produced in social interaction. Here, Davidson et al. (2014) have found that preschool children are expected to understand and produce institutional ways of talking about digital texts and experiences.

The touchscreen has made other symbols than the alphabet important when it comes to using digital technologies. This means that reading and writing in the traditional linguistic sense are not the only ways to work with such devices. Lately, touch has become interesting to literacy researchers (e.g., Bezemer and Kress, 2014; Crescenzi et al., 2014; Nacher et al., 2015; Price et al., 2015). Price et al. (2015) have conducted a comparative study of touch-based interaction where they investigate the use of tablets in comparison to using paper when drawing/painting, and have found differences in how children use their fingers. Crescenzi et al. (2014) point out that the properties of the environment have implications for the type of touch that children use and how they use touch. They argue that the interface shapes young children’s touch-based interaction. When the focus is on touch, what is interesting is how children deal with the interfaces, such as touchscreens, as well as the possible knowledge and skills that children develop through participating. Studies of young children and touch are intriguing because they show that embodied competence is a necessity for making use of touchscreens. However, these studies mainly focus on what children are able to do at a certain stage in their motoric development (Nacher et al., 2015; Price et al., 2015), thus they tend to approach touch as a question of individual and psychological development, not as a social activity (Aarsand and Bowden, 2019).

Interfaces such as the touchscreen require that the user has a *visual competence* in terms of interpreting, understanding and producing signs and symbols within a socio-cultural setting, a *tactile competence* in terms of touching, swiping and tapping, and an *audio competence* in terms of understanding and acting on verbal instructions and cues. In the present study of children’s digital literacy practices, the social interaction and the organization of categorization activities are in focus. The study of social interaction includes looking into different modalities, such as talk, pointing, gaze, intonation, and other embodied actions in the pragmatic sense of meaning making (e.g., Goodwin, 2013).

## Guided Participation and Professional Vision

To study how children become competent users of digital tools I explore how they take part in activities where such tools are an integral part of them. This means that my main interest is not digital media *per se*, but practices where children use them as tools. To investigate such practices I will use two theoretical concepts: ‘guided participation’ (Rogoff, 2003) and ‘professional vision’ (Goodwin, 1994).

### Guided Participation

Guided participation can be explained as ‘*varied ways* that children learn as they participate in and are guided by the values and practices of their cultural communities’ (emphasis in original) (Rogoff, 2003, pp. 283–284). Guided participation points out that competence is the outcome of participating in practices, here digital literacy practices in preschool, and where through talking, doing, and stance taking adults and peers display social and cultural references regarding how to use digital tools. The notion of guided participation includes interaction that is intended as instructional but also activities that go beyond intended instruction, such as teasing, assessing, and shaming. The social aspect underlined by guided participation actualizes such questions as what kind of participation, who, where, and when. What is a valid way of working with the tablet? How do children and adults demonstrate that they are digitally competent users in preschool?

### Professional Vision

To understand how people become qualified participants in different sociocultural practices it has been pointed out that we need to consider more than language in the linguistic sense. In his seminal paper ‘Professional Vision’ (1994), Charles Goodwin finds that the practice of seeing is the outcome of learning and being part of a community of practices. What we see when we look at a screen, watch a football match, or look at dirt on the ground differs depending on our experiences, education, occupation, age, position, gender, and so on. To identify an action, a symbol, a color or a particular shape as something specific is something that we learn. Goodwin uses the notion of ‘professional vision’ in studies of such professions as archeology and law enforcement to show how members of these professions have learnt to act in qualified ways by being part of a particular community of practices. He finds that archeologists use tools to identify certain colors in the dirt as proof of early settlements. Seen from an anthropological point of view, dirt is turned into an object of knowledge. ‘Through the progressive development of, and apprenticeship within, diverse epistemic ecologies, communities invest their members with the resources required to understand each other in just the ways that make possible the accomplishment of ongoing, situated action’ (Goodwin, 2013, p. 21). In short, we learn to look at things in culturally specific ways (Linell, 2009).

Similar to professionals, children in preschool have to learn a range of practices to be ‘qualified’ for school and society at large. This means that they participate in social practices where they have to identify, describe and act on phenomena in their surroundings in socially acceptable ways. This could be identifying someone as sad, or a sign acting as something that tells us what to do. The complexity of social life is transformed into categories that constitute how to be in preschool. According to Goodwin (1994, p. 606), ‘An event being seen, a relevant *object of knowledge*, emerges through the interplay between a *domain of scrutiny* and a set of *discursive practices* being deployed within a *specific activity’* (emphasis in original). An object of knowledge may be shapes, forms, colors and expressions, for instance, it could be a traffic sign or a traffic light. These signs do not have a meaning in themselves, rather this meaning emerges through the interplay between a domain of scrutiny, which may be an object, an image or a movement, and a set of discursive practices that helps one to divide the domain of scrutiny by highlighting a figure on a background in that particular activity. Moreover, when a driver, a police officer and a transport researcher look at signs and traffic lights, they will most likely see and describe them differently.

Goodwin (1994) introduces three key aspects of professional vision: coding, highlighting, and representation. ‘Coding’ points to how within a certain practice a particular way of interpreting what is seen is used. Often, this is done by means of classification or coding schemes that help the user to structure the perception, for instance, to turn an object into a circle instead of seeing it as a football, or to see a green light as a symbol that allows us to cross the street. The coding schemes can be a ‘standard’ used in similar situations that help us to identify certain objects of knowledge. It could be argued that coding schemes control perception by giving the green light, when it appears by the road together with a yellow and red light, a particular meaning. ‘Highlighting’ points to the process whereby the viewer distinguishes between the figure and background, when a certain act, object, shape, or color is identified and displayed as something specific. Highlighting also refers to making something stand out, or to put it somewhat differently, something is made visible. To locate features of the phenomenon in question we could for instance point at something, or draw a line to make a distinction between the figure and its background. This last step involves what Goodwin calls the ‘production and articulation of material representation.’ He points to the interface between talk, writing practices, and tool use when producing verbal as well as material articulations (drawings, images, diagrams, tables, applications, and so on). The production of representations can be seen as a process where participants display how to act in qualified ways (or not).

In the present paper, Goodwin (1994) idea of how professionals see their surroundings in appropriate ways will be used as an analytical tool to scrutinize how through participating in adult-led activities children are placed in situations where they learn to see, interpret and act on their surroundings. I will use the concept of professional vision to discover how through instructions, norms, and values children are guided to see, understand and act as competent members of their society, and how digital tools are part of these processes.

## Methodology

The present paper is part of the project *Digital Tools in Early Childhood Education and Care*. The data material consists of approximately 70 h of video recordings from three Norwegian preschools. During the fieldwork, two video cameras have been used, one camera followed an adult (the focus adult) during the day and one followed a child (the focus child). In total, 45 children and eight adults participated in the study. To avoid unnecessary focus on one particular child, we did not video-record the same child 2 days in a row. The recorded children were 5–6 years old and part of the ‘school-starter’ group, which means that they will be entering elementary school next fall.

I have selected two cases for analysis based on the following criteria: (1) there should be a reoccurring activity, (2) the participant constellations should differ (child–child and child–adult interaction), and (3) variation with regard to the digital tool used (tablets, smartboard, and smartphones). These criteria are seen as important for being able to say something about digital literacy practices in preschool by revealing variations when it comes to how these are socio-materially organized and accomplished *in situ*.

The main reason for choosing the present excerpts is that they show how digital literacy activities in ECEC vary in terms of social norms, digital tools, and social constellations. The excerpts have been transcribed according to conventions developed within conversation analysis (see Appendix A). Frame grabs are used to highlight analytically relevant embodied actions and the participants’ orientations to the material environment. Frame grabs where faces are visible have been blurred to protect the anonymity of the participants. As the participants are Norwegian speakers, the excerpts have been translated into English. When it comes to ethical considerations, written informed consent was obtained from the preschool teachers and from the parents of the children for the purposes of research participation, as well as for the publication of data and images. The children were continuously informed during the fieldwork about the research project and their right to decide whether they wanted to participate or not (cf. Aarsand and Forsberg, 2010). The project has been approved by the Norwegian Centre for Research Data with respect to research ethics. Pseudonyms have been used for all participants.

In the analysis, focus is on the interactional resources (talk, text, images, moving images, music, and so on) that children orient to, and how digital tools become an integral part of their literacy practices. The analytical focus is on how the participants establish different participation frameworks (Goffman, 1974; Goodwin and Goodwin, 2004) by addressing the following question: how do children participate in categorization practices in preschool where digital tools are used?

## Using Digital Tools in Categorization Activities

Norwegian preschool children come together across social class, gender, and ethnicity lines (Statistics Norway [SSB], 2018), and the preschool is an arena where society communicates norms, values and what is expected of children to learn and master at a particular age (The Norwegian Directorate for Education and Training [UDIR], 2017). A key part of social and cultural life is to understand signs and symbols. They tell us how to act, but are also tools we use to categorize information. Categorizations are activities where objects, ideas, and theories are grouped to be used for particular purposes. This could be differentiating and grouping animals as mammals, or humans as women and men. Categorization is about using symbols and labels in ways that help us to create and sustain socially organized ways of knowing, seeing, and acting upon the world (Goodwin, 1994). In preschool, children are expected to learn the meaning of signs and symbols by participating in activities where they are used and made relevant (cf. Kress, 2000; Rogoff, 2003).

The two examples in this article focus on how children participate in two different categorization activities in preschools using digital tools. In the first example, the focus will be on how digital cameras work in the process of identifying and communicating geometrical shapes. In the second sample, the focus is on how a smartboard with applications works in the process of identifying and categorizing feelings.

### Geometrical Seeing and Digital Cameras

In the Norwegian Framework plan for kindergarten (The Norwegian Directorate for Education and Training [UDIR], 2017), one of the learning areas is entitled ‘Quantities, spaces, and shapes.’ This area covers ‘play and investigation involving comparison, sorting, placement, orientation, visualization, shapes, patterns, numbers, counting, and measuring’ (The Norwegian Directorate for Education and Training [UDIR], 2017, p. 53). In the first example, I will examine how children participate in the categorization of geometrical shapes, which are sociocultural artifacts produced and sustained through this particular field within mathematics, by using a digital camera. Geometry can be seen as an established way of thinking, viewing and understanding our surroundings.

#### Demonstrating Geometrical Seeing

The teacher has just shown the children what geometrical shapes look like by holding up sheets of paper with different shapes, a circle, a rectangle, a square and a triangle, while also telling them the name of each shape. The children have been divided into groups of two where they are given a tablet or a smartphone and are told to take pictures of geometrical shapes in the classroom. When we enter the first excerpt for this example, Stefan and June are standing in the middle of the room looking around.

**Table:** 

Excerpt 1a		
Participants^∗^: STEFAN, NOAH, JUNE, and Jon (preschool teacher)		
1	STEFAN	((Passes the tablet to Jon))
2	Jon	>Should I take a picture?<
3	STEFAN	Ye:s.
4	NOAH	Yes (.) I’ll be jumping on xxx first ((jumps around in the room))
5	Jon	Okay then I’ll take a picture (.) so what is a good motif a:::: what do I want to take a picture of? ((looks around in the room))>the clock<yes (.) what shape does the (.) June? ((looks at June))
6	JUNE	((turns toward Jon))
7	Jon	June look at the clock? ((pointing towards the clock with the tablet))
8	JUNE	((turns to the clock))
9	Jon	>Should we take a picture of the clock?< (3.0) ((Jon takes a picture of the clock with the tablet))
10	Jon	What shape is the clock? ((June walks away))
11	NOAH	Circle ((points at the clock on the wall))
12	Jon	Yes.
13		(2.0)
14	Jon	How is the picture? ((looks at the tablet)
15	STEFAN	Oh↑ it’s kind of dark.
16	Jon	Should we try again?
^∗^Written and informed consent was obtained from the adult and the parents of the children for publication of transcriptions of discourse data.		

Stefan passes the tablet to Jon (line 1), who takes it and asks if he wants him to take a picture. Jon accepts the request and starts talking out loud about what he is doing. In this way he draws attention to what he is doing, taking a picture, and how he does it. He has to find a good motif that fits the task given to the children and draws attention to ‘the clock’ (line 5). However, identifying a good motif is not enough, it also needs to be categorized as having a certain shape. Here, they are supposed to use the coding schemes that were introduced to them before they started this activity. Jon asks what the shape of the clock is, but June does not seem to focus on Jon’s demonstration, who is looking at June and addressing her (line 5). Jon addresses June once more, points toward the clock with the tablet in his hands and establishes a joint focus of attention (line 7). He then highlights the clock as a relevant object; it becomes an object that is transformed into a circle. This is even underlined by the fact that Jon takes a picture of the clock. Before the demonstration is finished, Jon asks for the name of the shape and Noah answers ‘circle’ (line 11). The answer is confirmed by the teacher before he looks at the picture on the tablet and shows it to Stefan who concludes that it is ‘kind of dark’ (line 18). Put briefly, Jon demonstrates that identifying geometrical shapes is not enough, they should also be named correctly and documented as visible on the device. Asking ‘how is the picture’ is an invitation to assess the ‘visual articulation’ of the shape, the picture. Being too dark may be an argument for taking a new picture. The digital camera makes it possible to create and recreate a visual articulation until one is satisfied. Moreover, Jon demonstrates and establishes a procedure that tells the children how to solve the task and how this includes using the digital camera correctly.

#### Making Visual Articulations

The children have been told what different geometrical shapes look like and the preschool teacher has demonstrated how to identify and highlight these shapes in the classroom. Part of this work concerns the production and articulation of a representation, the image. In the next excerpt, the children’s attention will be on how they produce a representation of geometrical shapes. Ida and Nils are standing in front of a table with several objects on it.

**Table:** 

Excerpt 1b		
Participants^∗^: IDA and NILS		
1	IDA	There↑ ((points))
2	NILS	Yes ((pulls out a box))
3	IDA	(2.0) ((tries to take a picture of the box))
4	NILS	O:h↑ g go back a bit
5	IDA	((Moves backwards with the camera pointed at the box))
6	NILS	Like that (.) that was nice
7	IDA	((Takes a picture))
8	IDA	The hou↑se ((points the camera at the house next to the box))
9	NILS	The house
10	IDA	(7.0) ((focuses with the camera on a Lego house))
11	IDA	Uhm:
12	NILS	Like that (1.0) let me see ((takes the mobile phone))
13	IDA	((Ida looks around in the room)) Oh this↑
14		(2.0) ((Ida runs to a shelf and takes down the object))
15	NILS	°Wait then wait then° ((turns the camera to the object))
16		(1.0)
17	NILS	Yes ye↑s it’s the peak that I made
18	IDA	(2.0) ((turns the pyramid to Nils))
19	NILS	A square
20	IDA	°Take a picture°
^∗^Written and informed consent was obtained from the parents of the children for publication of transcriptions of discourse data.		

Ida points at a box simultaneously as she says ‘there↑’ (line 1) indicating that she has identified something that they are looking for. Nils agrees with Ida and pulls the box out and arranges it on the table (line 2), which can be seen as a way of highlighting the geometrical shape of the object. This arrangement makes it easier to see and take a picture of the geometrical shape. Ida tries to take a picture of the box with the smartphone. Here, both Ida and Nils look at the object (the box) through the screen on the smartphone. The presumed picture is not satisfying and Nils asks her to move back a bit, which she does. By backing a few steps she is able to get the whole box on the screen. During this sequence, Nils looks at the screen and approves of Ida’s use of the camera ‘like that (.) that was nice’ (line 6), before she finally takes the picture. Moreover, in concert, they coordinate their bodies and the camera to create a representation of the object.

When they move to the next object, we see the same procedure once more; Ida identifies a possible shape and directs their attention to it by saying the name out loud, ‘the hou↑se’ simultaneously as she points the camera at it (line 8). Nils responds by repeating ‘the house’ (line 9) and thereby confirms that they have a joint focus of attention and an agreement of what has a valid shape. Both are looking at the screen at the same time as Ida works on getting the object in focus before she takes a picture (line 10). When this has been achieved, Nils aligns once more with Ida’s choice to photograph the house by saying ‘like that’ before he asks to see the picture (line 12).

Ida gazes around the room before her attention is drawn to another object (line 13), a Lego pyramid. She moves over to the shelf and takes the object down. Nils has not yet seen the object, but moves over to the shelf with the camera and points it at the object (line 15). During this sequence, Nils looks at the object through the screen and identifies it is as ‘the peak’ that he made (line 17). Ida arranges the object in a way that makes Nils see the pyramid from above and he says ‘a square’ (line 19). This is the first time that they actually verbally articulate what they see using a geometrical term. Usually, there are several potential geometrical shapes present in one and the same object. In this case, they could have chosen to highlight a triangle, however, Nils refers to what he sees *through* the smartphone, which is a square.

This excerpt shows how the children use geometrical shapes as a code to highlight the geometrical shapes in the objects they see. The digital camera is an essential tool when creating representations of the geometrical shapes. To make a representation they have to identify and highlight it in a way that makes it possible to see a figure on a background, and they have to be able to articulate and communicate this figure. To do this, they need to create a representation that is both visible and representative of the particular geometrical shape that they want to display. Taking pictures of the object is one way of articulating the geometrical shapes, and this can be seen as visual articulation. The children display clear ideas about what a good ‘visual articulation’ of the object looks like, how to arrange the object so that the whole shape appears on the screen and how to take a good a clear picture. Furthermore, we can also see how the children view the object through the screen from the very beginning, making it fit within the digital format that the camera suggests. Moreover, the main challenge seems to refer to being able to visually articulate the shape. Here, the camera is a tool that makes visual articulation possible but it also restricts what can be turned into an example of a geometrical shape. Being successful in the categorization of geometrical shapes indirectly becomes a question of knowing how to use the digital camera.

#### Assessing Articulation/Categorization

Identifying and visually articulating the different geometrical shapes is an important part of learning geometry, but this is not enough. It is also important to name and articulate the shapes verbally.

After the children have walked around in the preschool taking pictures of different shapes, they are gathered around a table in a corner of the room. The preschool teachers have collected the devices (smartphones and tablets) and they are now looking through the pictures that the children have taken. They are seated around a table on small chairs while the teachers are seated at the head of the table.

**Table:** 

Excerpt 1c		
Participants^∗^: STEFAN, JUNE, and the preschool teachers: Sara and Marte		
1	Sara	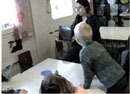 June have you taken this one? ((picture of a computer screen))
2	JUNE	E:: yes
3	Sara	What shape is it?
4	JUNE	M::::::: it’s a square
5	Marte	Then (.) what kind of square?
6	JUNE	Like this ((shows a paper sheet with the shape of a rectangle))
7	Marte	And what do we call it?
8	STEFAN	TRI: [ANGEL ((from under the table))
9	JUNE	[°Rectangle°
10	Marte	Recta[ngle yes
11	Sara	[Rectangle yes ((changes picture on the tablet)) and the last shape you have is this one
^∗^Written and informed consent was obtained from the adults and the parents of the children for publication of transcriptions of discourse data and images.		

Sara holds up the tablet and shows a picture of a computer screen to the group while simultaneously asking June if she is the one who has taken the picture. By addressing June, she makes her potentially responsible for the picture. By showing the picture to the whole group, Sara has established a joint focus of attention where she tells the children that this is what they will be talking about now. June confirms that she has taken the picture, which is followed by Sara asking her what shape the object is (line 3). June answers that the object in the picture is the shape of a square (line 4). This is not exactly the answer that Sara was looking for so Marte (another preschool teacher) specifies the question by asking what kind of square, thereby telling the children that there are different types. June holds up a piece of paper that has the shape of a rectangle and says ‘like this’ (line 6). This piece of paper was used when the preschool teachers introduced the children to the task. But, categorizing the square by saying ‘like this’ (line 6), even when she displays a piece of paper with the correct shape, is not considered good enough. The child also needs to name it correctly (line 7). When June finally says quietly ‘rectangle’ (line 9), both Marte and Sara confirm that she has given the correct answer.

The whole sequence can be seen as an assessment of June’s competence with respect to geometrical shapes. She is expected to demonstrate through visual and verbal articulation that she is able to identify and present a digital representation of the shape. In addition to this, she is expected to be able to talk about shapes using correct geometrical terms. Public assessment like this can be seen as guided participation where the children observe how the preschool teacher talks to their peers about different squares, triangles and circles, and the naming of these, about what is satisfactory visual articulation and what is considered to be a picture that is suitable for discussing in these terms. Moreover, the norm of how to use digital cameras and what is considered to be a good enough picture is communicated through public assessment and the joint visible focus of attention.

### Categorizing Thoughts and Feelings Using the Smartboard

In the next example, the focus is on how this preschool works on developing children’s social competences. It could be argued that social competence is something that people learn by being together with others, participating in social activities, such as play, and dealing with social expectations (cf. Hutchby and Morran-Ellis, 1998). The focus here is on how preschool children participate in categorization of feelings by looking at images and drawings of children in various situations on a smartboard in a teacher-led activity. The application ‘Green thoughts – happy children’ that is used here is described as a ‘psychological first-aid kit’ that claims to train and stimulate children in how to talk about thoughts and feelings (Raknes, 2014). The application is part of the learning resource ‘Salaby’^1^.

#### Differing Between Red and Green Thoughts

In the first excerpt, the analytical gaze will be directed at how the preschool teacher introduces the codes ‘green’ and ‘red’ to label thoughts. The group consists of seven children and two adults. They are located in a room with a table, a smartboard in the front and a computer that is connected to it. The preschool teacher is seated next to the smartboard in front of the computer while the children are seated around the table. The lights are turned off, the door to the corridor is closed. They have just started the ‘Green thoughts – happy children’ application and the preschool teacher has told the children that they will be entering a preschool called ‘Anthill.’

**Table:** 

Excerpt 2a		
Participants^∗^: SOFIE, EMIL, Liz (preschool teacher), and sb (smartboard)		
1	Liz	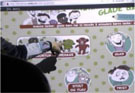 ((Points at the green teddy bear on the screen)) do you see the green teddy bear?
2	Xxx	Yes ((the children in chorus))=
3	Liz	=Yes (0.5) I believe that he’s sort of ha:ppy thoughts
4		(2.0) ((Liz moves the pointing finger to the red teddy bear))=
5	EMIL	=That one is not having nice thoughts
6	Liz	No he’s a bit red ((makes circles around the red teddy bear)) so maybe he is red thoughts ((moves the finger to the green teddy bear)) green thoughts and ((moves the pointing finger to the red teddy bear)) red thoughts. We will visit them in Anthill preschool ((starting a film))
^∗^Written and informed consent was obtained from the adult and the parents of the children for publication of transcriptions of discourse data and images.		

The preschool teacher directs the children’s attention to a drawing of a smiling green teddy bear by pointing at it and asking if they see it (line 1), thereby establishing a joint focus of attention. The children answer in chorus that they have identified it and thereby confirm this. Then the preschool teacher says: ‘I believe that he’s sort of ha:ppy thoughts’ (line 3), thereby relating the green teddy bear to happy thoughts. However, using the word ‘believe’ makes this symbol ambivalent and indicates that it could be interpreted differently. Then the preschool teacher slowly moves her index finger over to a red teddy bear (with a regular face), and Emil immediately claims ‘that one is not having nice thoughts’ (line 5). The preschool teacher approves Emil’s statement by saying ‘no he’s a bit red’ (line 6) at the same time as she draws a circle around the red teddy bear and highlights which one they are talking about. Thus, she confirms that there is a connection between the red teddy bear and not having nice thoughts. The relation is not made explicit by the preschool teacher, in fact she says ‘maybe he is red thoughts’ (line 6), but what these thoughts are is not described nor talked about. However, by pointing at, circling in and pointing out the color of the teddy bear that she is talking about, she introduces what could be called a *visual code.* This code consists of two distinct colors, green and red, which represent two categories of thoughts, ‘happy’ and ‘not so nice.’ When looking at pictures of children in interaction in the upcoming part of the application, these two possibilities can be identified and highlighted with this visual code. Using the code, the children have to choose just one of the categories when describing the thoughts/feelings of the person(s) in the picture.

#### Identifying and Categorizing Feelings

After the children have been introduced to the ‘visual code’ and have watched an animated film about the preschool called Anthill, they are invited to go up to the smartboard one at a time to solve the task: ‘How do the children feel?’ The task is to connect a particular feeling to a graphic representation, a drawing of a child. The children have to solve the task in front of the class, which means that how they deal with the task is visible to the entire group. In terms of learning, this can be seen as demonstrating to the rest of the class how to identify and label feelings.

In Excerpt 2b, Anne has come up to the smartboard and the teacher has started the application. First, the computer names a feeling, second, Anne has to choose one out of two drawings of a person who symbolizes this feeling, and finally, the computer assesses Anne’s answers. The task is to identify the ‘correct’ representation. It is important to note that there is a technical glitch with the smartboard; it does not respond to touch on its icons and symbols. For this reason, the teacher uses the mouse on the computer to maneuvre on the touchscreen.

**Table:** 

Excerpt 2b		
Participants^∗^: ANNE and Liz (pre-school teacher) and sb (smartboard)		
1	Sb	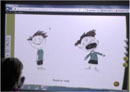 Roald is scared ((two drawings appear on the screen))
2		(2.0)
3	ANNE	((Points at the drawing to the right))
4		(3.0)
5	Liz	°Yes↑° ((Points with the mouse and clicks on the drawing to the right))
6	ANNE	((Takes away her pointing finger))
7	sb	Scared ((a third smiling face appears on the right side of the screen))
8	Liz	°Good Anne°
9	sb	Roald is sad ((two drawings appear on the screen))
10		(1.0)
11	ANNE	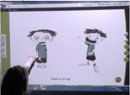 ((Points at the drawing to the left))
12	Liz	((Points with the mouse and clicks on the drawing to the left))
13	sb	Sad ((the fourth smiling face appears on the right side of the screen))
14		(2.0)
15	Sb	Trine is proud ((two drawings appear on the screen))
16	ANNE	°Hi:hi°
17	ANNE	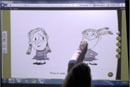 ((Points at the drawing to the right))
18	Liz	((Points with the mouse and clicks on the drawing on the right side))
19	sb	Proud ((a fifth smiling face appears on the right side of the screen))
20		(1.0)
21	sb	((Green teddy bear appears on the screen and music is playing)) Hurray↑
		now you were clever
22		(2.0)
23	Liz	Good Anne
^∗^Written and informed consent was obtained from the adult and the parents of the child for publication of transcriptions of discourse data and images.		

Two drawings appear on the screen and the smartboard states out loud ‘Roald is scared’ (line 1). Anne starts by looking at both pictures before she points at the picture to the right and touches the screen. In fact, Anne keeps her finger on the touchscreen until the preschool teacher moves the cursor to the drawing and clicks on it (lines 3–4). Thus, she demonstrates that she knows how the touchscreen works and that she has to keep her finger on the screen until the teacher clicks on the drawing because the screen does not work as it should. By keeping her finger on the screen, she makes sure that the preschool teacher sees and clicks on the drawing she has pointed to. After the preschool teacher has clicked on the drawing, the smartboard says ‘scared’ simultaneously as it assesses the answer (line 7). The assessment appears as a smiling face that pops up on the right-hand side of the screen. The screen shows us that she has a total of three smiling faces. The assessment is followed by the preschool teacher, who lowers her voice and aligns with the application (line 8).

In the excerpt here the same procedure occurs twice more. The child is presented to a feeling, scared (line 1), sad (line 9), and proud (line 15). Each time, the child is given two images to choose between, either X or Y, followed by an assessment made by the application, given as a smiling face. When the task has been fulfilled and Anne has collected five smiling faces, a dancing green teddy bear appears on the screen simultaneously as we hear a voice saying ‘Hurray↑ now you were clever’ (line 21). As we remember from the introduction to the ‘Green thoughts – happy children’ application, the green teddy bear represents happy thoughts, thereby recycling the assessment displayed by the smiling faces. The preschool teacher aligns with this assessment as well.

All in all, we can see how the application structures the activity following an IRE (initiative-response-evaluation) pattern (cf. Mehan, 1979). It starts by addressing the child, then waiting for an answer where the child chooses between two predefined images and then assessing the answer. The question of identifying feelings becomes a question where the child has an either/or option. The tool thus also restricts which feelings can be talked about, and even presumes that the feeling in question, for instance ‘proud,’ has a universal template. The feeling is not turned into a question of highlighting what makes it different from the others, nor does the child need to display how to use the concepts that describe emotions. During this sequence, the preschool teacher only gives minimal responses to Anne, which can be seen as an alignment with how the application accomplishes the activity. On a speculative note, it could be critically discussed whether or not the application restricts how to identify, articulate, and talk about feelings in preschool.

#### Assessment and Categorization Trouble

The smartboard obviously matters when it comes to how children learn to see, categorize and articulate feelings. The excerpt above shows us that the design of the application restricted a possible discussion of feelings because it presented predefined templates where the children were to choose one of two options.

The ‘green thoughts – happy children’ application has several types of tasks and I will now turn my focus to how Stefan tries to solve the task ‘My feelings.’ The task is structured into two parts. First, ‘when someone does X to you, how do you feel?’ which should be categorized as either a green or a red feeling. Second, when the child has chosen green or red, s/he has to rate the strength of this feeling on a four-grade scale. In the next excerpt we will see how Stefan gets into trouble when he is asked to categorize feelings.

**Table:** 

Excerpt 2c		
Participants^∗^: STEFAN, ANNE, Karen (preschool teacher), and sb (smartboard)		
1	Sb	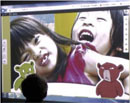
		When somebody pulls my hair?
2		(2.3)
3	Karen	What feeling do we get then? When somebody pulls our hair?
4	STEFAN	((Walks up to the smartboard)) that one ((and points to the red teddy bear)) no that one ((points to the green one))
5	Karen	Do you get happy?
6	STEFAN	Mm:: ((turns to the red teddy bear)) no: ((points to the red teddy bear and looks at the preschool teacher))
7	Karen	Sad?
8	STEFAN	Yes =
9	Sb	**Angry!**
10	ANNE	Angry.
11	Stefan	Ye::s
12	KAREN	How angry do you get? ((Points with the mouse on the screen))
13	Sb	Angry (.) very angry (.) quite angry (.) a bit angry?
14	STEFAN	Uh:::quite angry ((presses on the drawing in the middle of the screen))
^∗^Written and informed consent was obtained from the adult and the parents of the child for publication of transcriptions of discourse data and images.		

The computer asks: ‘when somebody pulls my hair’ (line 1). Stefan looks at the screen but does not answer. After a rather long pause, 2.3 s, the preschool teacher reformulates the question and asks: what feeling do we get when somebody pulls our hair? Stefan walks up to the smartboard and points first at the red teddy bear before he changes his mind and points to the green one. The preschool teacher’s assessment is delivered as a question in which she asks if he becomes happy. According to the preschool teacher, Stefan did not use the visual code to highlight and categorize the feeling correctly the first time. This leads Stefan to point to the red teddy bear once more while at the same time looking at the preschool teacher.

After Stefan has chosen the red teddy bear for the second time, the preschool teacher suggests that he feels ‘sad,’ which is confirmed by Stefan (line 8). It could be argued that the opposite of happy is sad. Thus, a dichotomy has been established, if you are not happy then you are sad. The preschool teacher then presses the red teddy bear on the screen and the computer says loudly ‘angry’ (line 9). The label angry is repeated and confirmed by the teacher (line 10) and Stefan aligns with her (line 11). In this way the feelings sad and angry fall into the same category. According to the teacher’s reaction, it seems important to be able to categorize feelings in terms of green and red, but it does not seem important how the feelings are more precisely labeled; sadness and anger are both possible. Red and green as categories of feelings are in the best case approximate. Note that green and red were originally introduced as labels of thoughts, not labels of feelings (see Excerpt 2a).

What is seen in the episode above is that the category red, which in the introduction part of this lesson was explained as ‘not nice thoughts,’ embraces both the feelings sad and angry. It could be argued that the teacher approves that the categories red and green can be used on a range of different feelings and that several of these feelings are labeled as ‘not nice’ (Excerpt 2a). Moreover, it is added that being angry or sad is not nice. But being able to choose between red and green, the children also need to know how to work with the digital equipment. In the example, Stefan demonstrates that he knows that he has to listen to the instructions given by the application (audio competence), and that he has to press the screen to give an answer (visual and tactile competence). He even knows how to handle the complementary instructions given by the teacher (social norms and expectations). In contrast to highlighting and categorization of geometrical shapes using digital cameras in peer groups, here we see how there is restricted room for discussions on how to highlight the feeling due to the establishment of the dichotomy and the immediate assessment.

## Digital Literacies in the Everyday Life of Preschool Children

Knowing how to work with digital tools is taken for granted in a wide range of activities in the everyday life of children. In the present study, I have used the notion of guided participation and professional vision to scrutinize the use of digital cameras and smartboards to see how these taken-for-granted technologies are tools that transform and influence how children learn to think and act as members of a particular socio-cultural practice. By examining the categorization practice, I have shown how children are introduced to two different symbol systems, representing geometrical shapes and feelings (thoughts), in ways that create and sustain socially organized ways of knowing, seeing, and acting upon the world (cf. Goodwin, 1994). Being a preschool child means being able to participate in various digital literacy activities where the main purpose is not necessarily about working with digital tools (cf. Pink and Mackley, 2013), rather this competence becomes a condition for solving the primary task.

We have seen here how digital cameras and smartboards work as tools used by children who have been taught to apply symbol systems to describe and understand their surroundings, also called ‘professional’ visions. In the first example, the children worked on creating a visual representation using the digital camera. How to use the camera is taken for granted, no instruction is given and the children are responsible for solving this on their own. Here, we saw how they struggle to get the object in focus and we saw that this work was guided by social and cultural norms as to what a picture is supposed to look like in terms of light and distance. The cultural norms are produced and reproduced not only according to the teacher’s demonstration of how to highlight and visually articulate the geometrical forms, but also by cooperating on taking pictures and through public assessment where the participants looked at and talked about what they saw in the pictures. The children also displayed that the practice of taking pictures using a digital camera includes taking several pictures of the same object. Interestingly, when searching for possible objects for a picture, they viewed their surroundings through the screen on the camera (cell phone or tablet). Objects that were too small or too big to be seen on the screen were not considered as a potential geometrical shape.

In the second example, we saw how children dealt with categorization of feelings by solving tasks within the learning resource ‘Green thoughts – happy children.’ In this example as well, how to deal with digital technology is taken for granted. The instructions are given verbally by the application and the child presses the icons and symbols on the smartboard. Because of a technical glitch, the children even coordinated their choices with the preschool teacher who had to complete them by means of the computer. Immediately after the children had decided to categorize a feeling and pressed the symbol, they received feedback. The way these applications are constructed, there are only two options and only one ‘correct’ answer. The children proved themselves to be competent users of smartboards and applications like ‘Green thoughts – happy children.’ However, the digital tool as it is used in the preschool practice becomes a matter of turning feelings/thoughts into an either/or question. Even though there seems to be disagreement between the child, preschool teacher and the application, there is no discussion that moves beyond the either/or question.

Using digital cameras and smartboards also means that bodily actions are important. In contrast to studies on touchscreens that mainly have focused on what children are able to do at a certain stage in their motoric development (Nacher et al., 2015; Price et al., 2015), the present study has shown how touch is just one among several embodied actions that are used in the social organization of digital literacy activities simultaneously. This can then be seen as an argument for a multimodal approach when investigating such activities. We saw how the children have to physically highlight the figure that they want to photograph, and they have to adjust their position with the camera to the situation at hand to find the best way of taking the picture. We also saw that the body is important in using the smartboard. As users, the children had to locate themselves to see the whole screen, they had to be able to differentiate between pointing at and pressing a symbol and, in the particular case that we witnessed, they had to coordinate their bodily actions with the teacher who then completed their choices.

All in all, categorization practices in preschools deal with symbols and labels in ways that help children to create and sustain socially organized ways of knowing, seeing, and acting upon the world. Digital media are embedded in routines, procedures, and socialites that are part of these categorization practices, they are part of how we teach children to experience, interpret, understand, and act in the world. Then, digital literacy can be seen as a pragmatic resource learned and used as children participate in everyday activities and where digital tools are inseparable from these. However, different technologies created different conditions for the children’s participation. Peer interaction was part of the digital literacy activities that involved such mobile technologies as smartphones and tablets (cf. Danby et al., 2018), while when using non-mobile technologies, like smartboards, it is shown that the activities were structured more as ‘classic’ classroom activities, primarily guided by the teacher and the didactic material presented through the smartboard.

Taking an ethnomethodological/conversation analytical approach to digital literacy activities *in situ* yields new understanding of social interactions when using digital tools in everyday activities in Early Childhood Education and Care. The detailed analysis displays how children develop their ‘professional’ vision through such social activities as categorization practices where they adjust their action to norms and expectations (cf. Davidson et al., 2014, 2017). The social organization of the categorization activities was partly related to the digital tools that were used. Collaboration and social interaction were an important part of solving the task they faced. While this is not a new finding (e.g., Björk-Willén and Aronsson, 2014; Danby et al., 2018), the present paper’s findings show how digital tools are integrated in the creation of knowing and socially organized ways of seeing and understanding, and that digital tools are not neutral and non-ideological mediators in these processes.

## Ethics Statement

The project has been approved by the Norwegian Center for Research Data.

Informed consent has been retrieved from staff in the preschool and the participating children’s parents. They have got written information about the project, the purpose and how this will influence on their children’s life in the preschool while doing the fieldwork. They have been informed of the possibility to withdraw at any moment from the project without any consequences. The children have been orally informed about the project and were given the opportunity to say no if they did not want to participate. Since we see consent as something that is (re)negotiated in the meeting with the children during our fieldwork, we have avoided video recording of children that in one way or the other signalized that they did not feel comfortable being observed.

## Author Contributions

The author confirms being the sole contributor of this work and has approved it for publication.

## Conflict of Interest Statement

The author declares that the research was conducted in the absence of any commercial or financial relationships that could be construed as a potential conflict of interest.

## Footnotes

1 www.salaby.no

## Appendix A

**Table A1:** Transcription conventions adapted from Jefferson (2004).

=	Equal signs indicate no break or gap between the lines.
(0.8) (.)	Numbers in parentheses indicate silence. A dot in parentheses indicates a micropause less than 5/10 of a second.
., ?	The punctuation marks indicate intonation. The period indicates falling intonation, the comma continuing intonation and the question mark rising intonation.
::	Colons are used to indicate prolongation or stretching of the immediately prior sound.
word Word	Underlining indicates some form of stress or emphasis. The more the underlining the greater the emphasis. Especially loud talk is indicated by bold font.
↑	The up arrow marks a sharp rise in pitch.
> <	Right/left carats indicate that the talk between them is sped up.
(())	Double parentheses are used to mark the transcriber’s descriptions of events.
JUNE	Full name written in capitals indicates that the person is a child.
°talk°	The degree signs indicate that the talk between them is quieter than surrounding talk.
